# Actinomycosis

**Published:** 1901-03

**Authors:** 


### ACTINOMYCOSIS.

Porter (*Boston Medical and Surgical Journal,* No. 11, 1900) states that the object of his paper is to attract attention to the possibility that a proportion of the cases ranking as alveolar abscesses may be due to this specific organism.

Though actinomycosis has been considered a relatively rare disease, he notes that it seems more probable that it is one which is very commonly overlooked. In its clinical aspects there is little that is characteristic. Though the course of the infection may make the surgeon suspicious, examination by microscope and culture is essential for a positive diagnosis.

The infection seems to enter most frequently near a carious tooth, or is carried in by a foreign body through the mucous membrane of the mouth or pharynx. Infection is rarely pure, but is usually mixed with ordinary pyogenic organisms or mouth bacteria. Clinically and under the microscope the disease is characterized by the formation of an unusual amount of dense connective tissue, which ends more or less abruptly at the periphery and infiltrates the adjacent muscle or fat. In the jaw the bone itself is rarely involved in human actinomycosis, though it may be thickened from periostitis.

Glandular enlargement is conspicuous by its absence, and when present seems to be due to mixed infection. Metastasis seems to occur through the blood-current and not by way of the lymphatics. In serious cases the disease may progress down the neck, into the antrum, or through the base of the skull.

Trismus, though often present, is no more characteristic of this disease than of other inflammatory affections, though if the masseter were involved in the dense connective tissue, the jaw would probably remain stiff for a long time.

It is rarely possible, the author states, to make a clinical diagnosis of actinomycosis; recurrent abscesses without necrosis, chronic, painless, subcutaneous abscesses about the jaw, evidently not connected with tubercular glands, would lead to a suspicion of this disease. If these fluctuating areas were surrounded by especially firm and hard connective tissue, and a sinus could be felt under the skin, if there were little œdema and swelling, perhaps a probable diagnosis could be made.

Examination of the discharge is of great assistance, but the mere presence of the so-called sulphur granules is not conclusive, and no case should be considered as one of actinomycosis without competent microscopic examination.

In examining for actinomycosis, gauze sponges which absorb the discharge should not be used. All bleeding, when possible, should be stopped before opening the abscess wall. Unless badly contaminated, actinomycosis pus appears usually as a clear, perhaps blood-tinged, slightly syrupy sero-pus. Placed on a cover-glass the granules vary in size from a millet-seed to the head of a large pin. They are usually round with a clear-cut periphery; the color is gray or grayish yellow, often suggesting a small pearl. The centre is not rarely somewhat darker. Fluid should be examined at once, for these granules are found with great difficulty when the blood has clotted.

With reference to treatment, the author thinks that two facts speak strongly for the self-limitation of the disease in the majority of cases. Though it cannot be a rare affection, few cases enter the hospital with advanced actinomycosis of the jaw, and it seems therefore certain that many recover after a simple incision of the abscess, and even through a natural rupture of it.

It is surprising to find, on microscopic examination of sections, how infrequently the colonies are found in the walls of the abscesses, though the pus contains many granules. The surrounding connective tissue probably proves an effective barrier to the spread of the disease.

Simple opening, curetting, and drainage have proved sufficient in many cases; though recurrences may be frequent, healing eventually takes place. Where possible, excision of the inner half of the abscess wall or sinus is the best treatment. The danger from swallowing the granules, where the discharge empties into the mouth, is hard to estimate. Certain cases of generalized disease in the lungs, intestinal tract, liver, etc., occur in which the organism gains entrance into the food, or is swallowed; and, therefore, the surgeon should aim at making external drainage.

The individual case and the severity of the infection must decide between curetting and cauterizing the cavity with tincture of iodine or carbolic acid and a more radical incision. Iodide of potash, in doses of twenty grains three or four times a day, has distinctly influenced some cases for good, and should be used in connection with the local treatment.—*The Therapeutic Gazette.*

